# Supplementary material for: Unraveling the rheology of inverse vulcanized polymers
Source: Nat Commun. 2023 Nov 20;14:7553. doi: 10.1038/s41467-023-43117-1 (PMC10662295; doi:10.1038/s41467-023-43117-1)
Supplement: Supplementary file 1 — Supplementary Information [file 41467_2023_43117_MOESM1_ESM.pdf]

Supplementary Information

Unraveling the Rheology of Inverse Vulcanized Polymers

Derek J. Bischoff[a], Taeheon Lee[b], Kyung-Seok Kang[b], Jake Molineux[b], Wallace O'Neil Parker, Jr.[c], Jeffrey Pyun[b]*, Michael E. Mackay[a,d]*

[a]Department of Materials Science and Engineering, University of Delaware, Newark, Delaware 19716, United States

[b]Department of Chemistry and Biochemistry & Wyant College of Optical Sciences, University of Arizona, Tucson, Arizona 85721, United States

[c]Physical Chemistry Department, Eni S.p.A., San Donato Milanese 20097, Italy

[d]Department of Chemical and Biomolecular Engineering, University of Delaware, Newark, Delaware 19716, United States

Supplementary Methods:

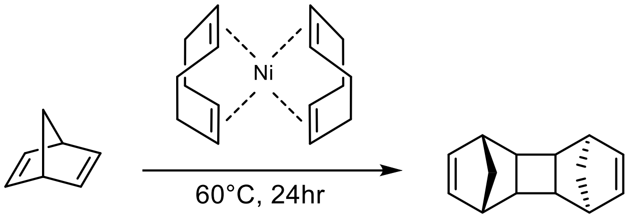

Synthesis of divinylic norbornadiene monomer NBD2: Bis(1,5-cyclooctadiene)nickel(0) (406 mg, 1.47 mmol) was added to a flame-dried Schlenk flask equipped with a magnetic stir bar under an argon atmosphere. The flask was vacuum purged and backfilled with argon three times before 2,5-norbornadiene (27.2 g, 30.0 mL, 295 mmol) was added, followed by 1,4-dioxane (30 mL). The reaction vessel was sealed and placed into a preheated oil bath at 60 °C for 24 hours. The reaction was then cooled to room temperature, and diluted with tetrahydrofuran (THF) (15 mL), and vacuum filtered. The filtrate was collected, and the solvent was removed via rotary evaporation to yield an off-white/tan solid. The crude product was further purified by sublimation at 80 °C/0.15 mmHg for 4 hours. The sublimation product was collected from a cold finger with dichloromethane (DCM), dried with magnesium sulfate, and filtered. Finally, the DCM was removed via rotary evaporation yield a white powder. NBD2 (13.67 g, 74.18 mmol, 50.3 %): ^{1}H NMR (500 MHz, $CDCl_3$) δ 1.23 (d, J = 8.9 Hz, 2H), 1.34 (s, 4H), 1.68 (m, 2H), 2.62 (p, J = 1.77 Hz, 4H), 6.01 (t, J = 1.81 Hz, 4H). ^{13}C NMR (126 MHz, $CDCl_3$) δ 39.81, 42.24, 44.22, 136.17.

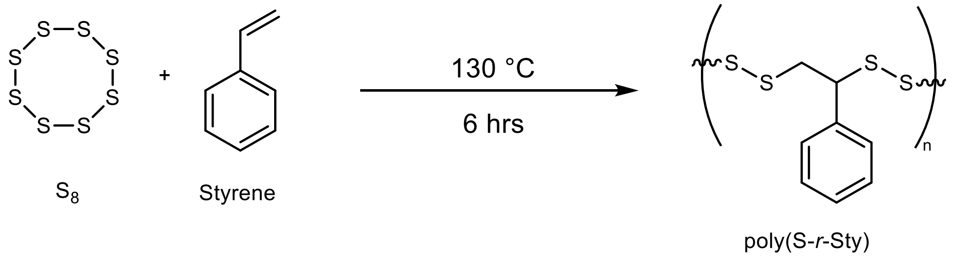

Copolymerization Procedure for Sulfur and Styrene - poly(S_{50}-r-Sty_{50}): To a 20 mL scintillation vial, elemental sulfur (2.5 g, 9.7 mmol) was added with a magnetic stir bar and heated to 135 °C in a thermostated oil bath until a clear yellow molten phase was formed. Uninhibited/purified styrene (2.5 g, 23.9 mmol) was added to the molten sulfur dropwise to prevent a sudden temperature drop. The polymerization proceeded for 6 hours at 130 °C until complete consumption of styrene was confirmed by ^{1}H NMR spectroscopy. Next, the reaction mixture was cooled to room temperature affording a viscous, red fluid. After the mixture was diluted in 10 mL of anhydrous THF, the solution was placed in a refrigerator overnight that induced precipitation of unreacted elemental sulfur which was removed by filtration. All volatiles were removed under reduced pressure and the polymer was dried under high vacuum overnight. (yield = 4.73 g, M_n = 1,100 g/mol, Đ = 1.24).

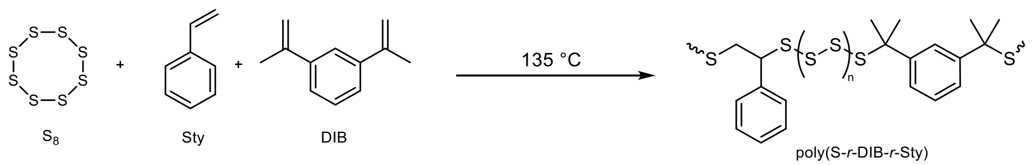

General Terpolymerization Procedure for Sulfur, DIB, and Sty –poly(S_{50}-r-DIB_{10}-r-Sty_{40}): To a 20 mL scintillation vial, elemental sulfur (2.5 g, 9.69 mmol) was added with a magnetic stir bar and heated to 135 °C in a thermostated oil bath until a clear yellow molten phase was formed. 1,3-Diisopropenylbenzene (DIB, 0.5 g, 3.16 mmol) was then injected into the molten phase via a syringe. Uninhibited/purified styrene (2 g, 19.1 mmol) was added next to the molten sulfur dropwise to prevent a sudden temperature drop. The polymerization proceeded until the viscosity prohibited continued mixing, after which the material cured at temperature for an additional hour. The reaction was cooled to room temperature before being cooled with liquid nitrogen to remove it from the vial for a quantitative yield.

Terpolymerization Procedure for Sulfur, DIB, and Sty – poly(S_{50}-r-DIB_{25}-r-Sty_{25}): The terpolymerization was carried out following the general method above using S_8 (2.5 g, 9.69 mmol), DIB (1.25 g, 7.89 mmol) and Sty (1.25 g, 11.95 mmol) to afford the polysulfide poly(S_{50}-r-DIB_{25}-r-Sty_{25}) with a quantitative yield.

Terpolymerization Procedure for Sulfur, DIB, and Sty –poly(S_{50}-r-DIB_{40}-r-Sty_{10}):
The terpolymerization was carried out following the general method above using S_8 (2.5 g, 9.69 mmol), DIB (2 g, 12.64 mmol), and Sty (0.5 g, 4.78 mmol) to afford the polysulfide poly(S_{50}-r-DIB_{40}-r-Sty_{10}) with a quantitative yield.

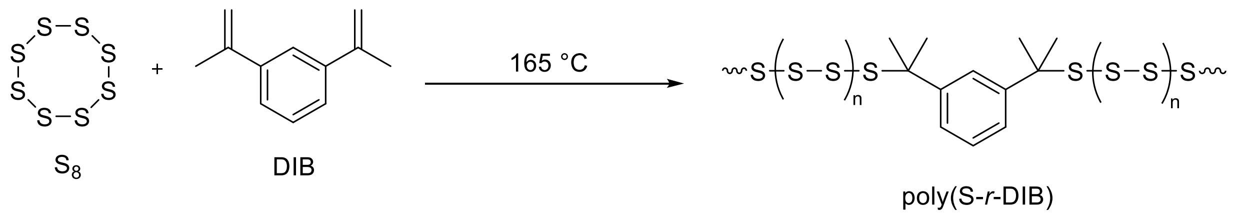

General Copolymerization Procedure for Sulfur and DIB - poly(S_{50}-r-DIB_{50}): Elemental sulfur (2.5g, 9.69 mmol) was added to a 20 mL glass vial equipped with a magnetic stir bar and was heated to 165 °C in an oil bath until a clear yellow molten phase was formed. 1,3-Diisopropenylbenzene (DIB, 2.5 g, 15.8 mmol) was then injected into the molten phase via a syringe. The resulting mixture was stirred at 165 °C for 8-10 minutes until stirring stopped due to the increased viscosity of the reaction mixture. After cooling to room temperature, the polysulfide was extracted from the vial to yield 4.89 g (97.8 %) of poly(S_{50}-r-DIB_{50}).

Copolymerization Procedure for Sulfur and DIB - poly(S_{70}-r-DIB_{30}): The copolymerization was carried out following the general method above using S_8 (3.50 g, 13.7 mmol) and DIB (1.50 g, 9.48 mmol) to afford the polysulfide poly(S_{70}-r-DIB_{30}) with a yield of 4.88 g (97.6 %).

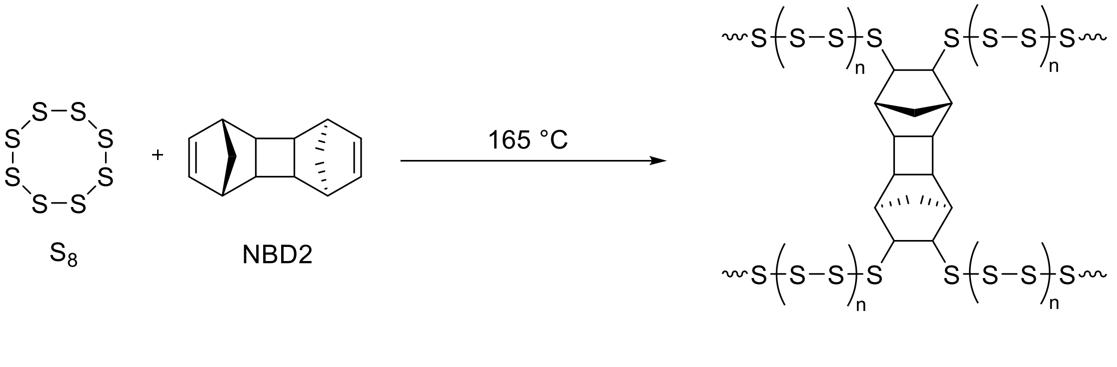

General Copolymerization Procedure for Sulfur and NBD2 - poly(S_{50}-r-NBD2$_{50}$): Elemental sulfur (2.5 g, 9.69 mmol) was added to a 20 mL glass vial equipped with a magnetic stir bar and was heated to 165 °C in an oil bath until a clear yellow molten phase was formed. Norbornadiene (NBD2, 2.5 g, 13.56 mmol) was then added to the molten phase. The resulting mixture was stirred at 165 °C for 15 minutes until stirring stopped due to the increased viscosity of the reaction mixture. After cooling to room temperature, the polysulfide was extracted from the vial to yield 4.91 g (98.2 %) of poly(S_{50}-r-NBD2$_{50}$).

Copolymerization Procedure for Sulfur and NBD2 - poly(S_{70}-r-NBD2$_{30}$): The copolymerization was carried out following the general method above using S_8 (3.50 g, 13.7 mmol) and NBD2 (1.50 g, 8.14 mmol) to afford the polysulfide poly(S_{70}-r-NBD2$_{30}$) with a yield of 4.90 g (98 %).

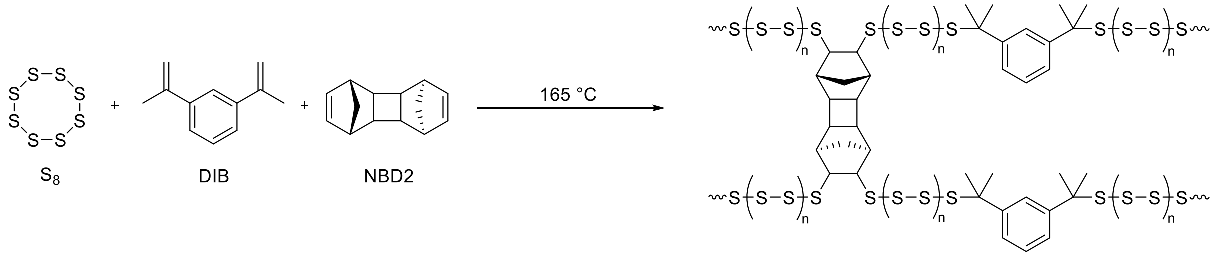

General Terpolymerization Procedure for Sulfur, DIB, and NBD2 - poly(S_{50}-r-DIB$_{10}$-r-NBD2$_{40}$): Elemental sulfur (2.5 g, 9.69 mmol) was added to a 20mL glass vial equipped with a magnetic stir bar and was heated to 165 °C in an oil bath until a clear yellow molten phase was formed. Norbornadiene (NBD2, 0.5 g, 2.71 mmol) was then added to the molten phase. 1,3-Diisopropenylbenzene (DIB, 2 g, 12.64 mmol) was then injected into the molten phase via a syringe. The resulting mixture was stirred at 165 °C for 15 minutes until stirring stopped due to the increased viscosity of the reaction mixture. After cooling to room temperature, the polysulfide was extracted from the vial to yield 4.91 g (98.2 %) of poly(S_{50}-r-DIB$_{10}$-r-NBD2$_{40}$).

Terpolymerization Procedure for Sulfur, DIB, and NBD2- poly(S_{70}-r-DIB$_{15}$-r-NBD2$_{15}$): The terpolymerization was carried out following the general method above using S_8 (3.50 g, 13.7

mmol), DIB (0.75 g, 4.74 mmol) and NBD2 (0.75 g, 4.07 mmol) to afford the polysulfide poly(S_{70}-r-DIB_{15}-r-$NBD2_{15}$) with a yield of 4.90 g (98 %).

Terpolymerization Procedure for Sulfur, DIB, and NBD2- poly(S_{50}-r-DIB_{25}-r-$NBD2_{25}$): The terpolymerization was carried out following the general method above using S_8 (2.50 g, 9.69 mmol), DIB (1.25 g, 7.89 mmol) and NBD2 (1.25 g, 6.78 mmol) to afford the polysulfide poly(S_{50}-r-DIB_{25}-r-$NBD2_{25}$) with a yield of 4.90 g (98 %).

Terpolymerization Procedure for Sulfur, DIB, and NBD2- poly(S_{50}-r-DIB_{40}-r-$NBD2_{10}$): The terpolymerization was carried out following the general method above using S_8 (2.50 g, 9.69 mmol), DIB (2 g, 12.64 mmol) and NBD2 (0.5 g, 2.71 mmol) to afford the polysulfide poly(S_{50}-r-DIB_{40}-r-$NBD2_{10}$) with a yield of 4.92 g (98.4 %).

Supplementary Data:

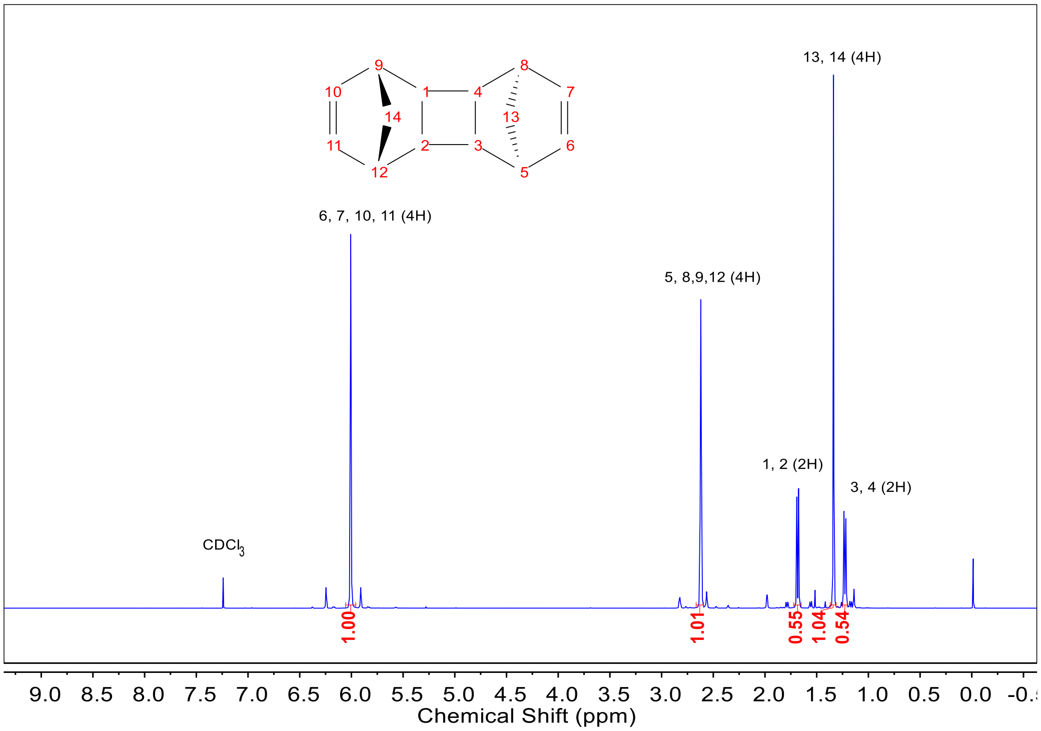

Supplementary Figure 1: ^{1}H NMR spectrum of synthesized NBD2 norbornadiene monomer. Material is dissolved in $CDCl_3$ with signal assignments using TMS as an internal standard at δ 0.

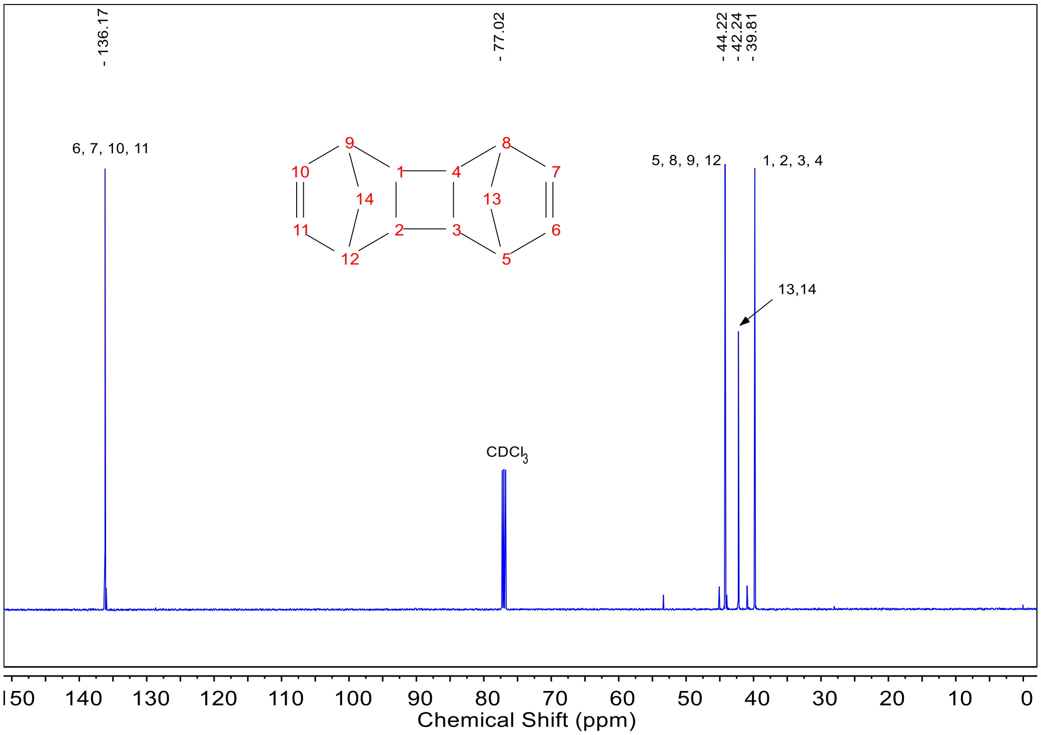

Supplementary Figure 2: ^{13}C NMR spectrum of synthesized NBD2 norbornadiene monomer. Material is dissolved in $CDCl_3$ with signal assignments using TMS as an internal standard at δ 0.

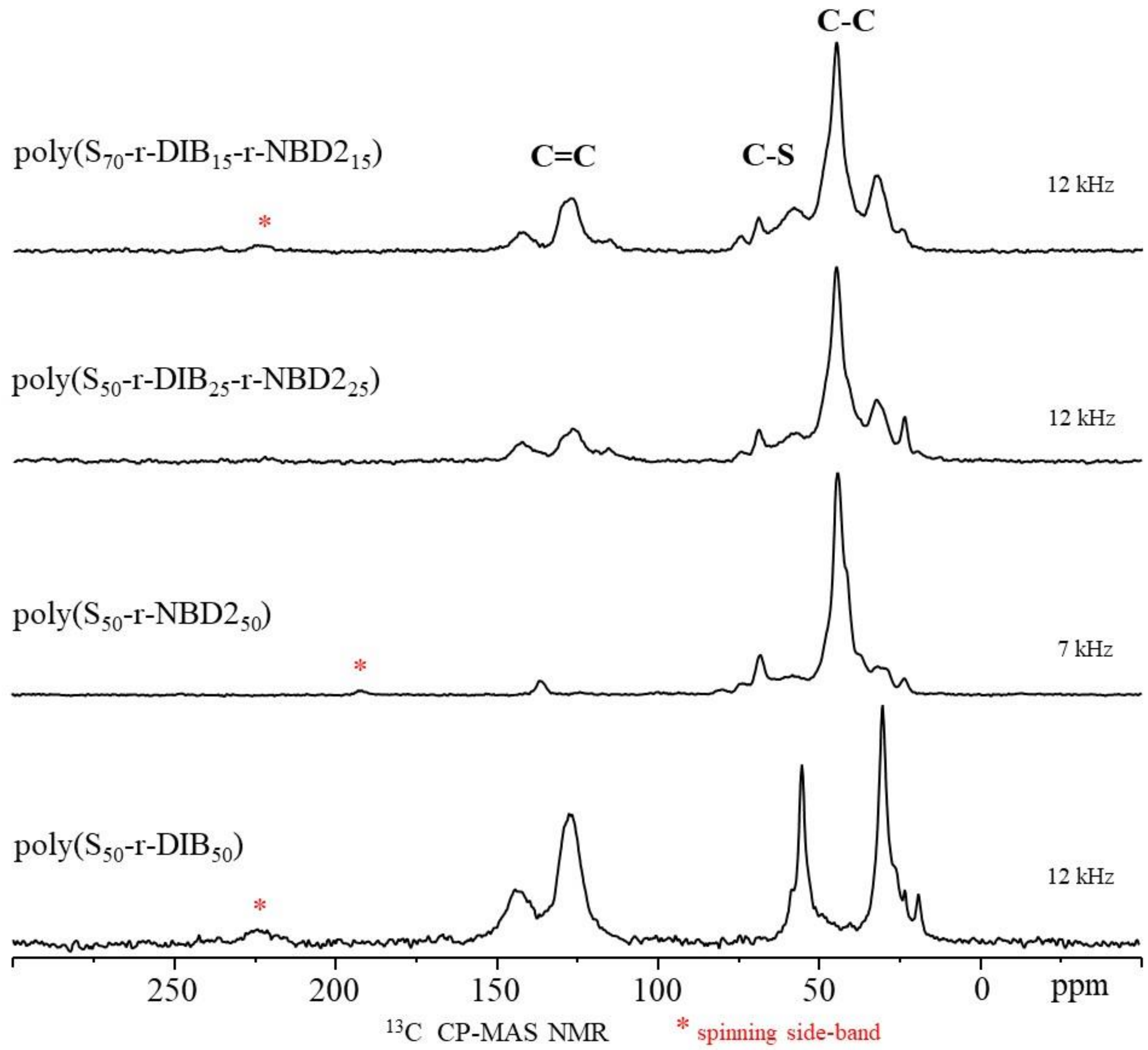

Supplementary Figure 3: ssNMR results. ^{13}C ssNMR spectra showing incorporation of organic DIB and NBD2 moieties in inverse vulcanized polymers.

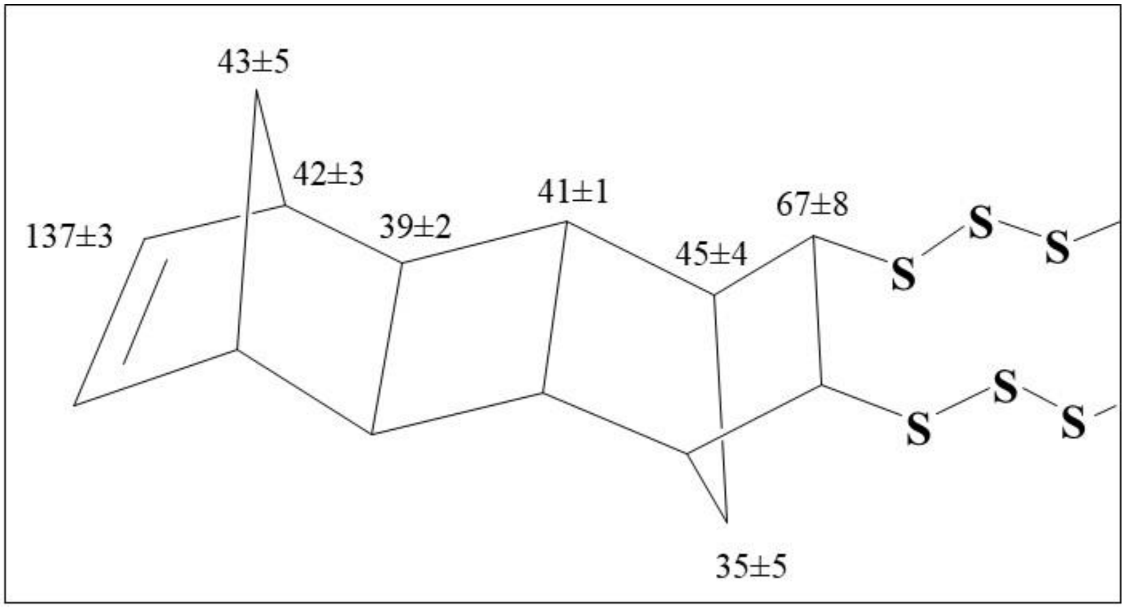

Supplementary Figure 4: ssNMR results. ^{13}C NMR shifts predicted for the partially reacted NBD2 monomer in poly(S-r-NBD2) using ACD 2018. The minor signal near 137 ppm observed in ^{13}C spectrum of previous figure is due to a small amount of unreacted NBD2 olefinic group.

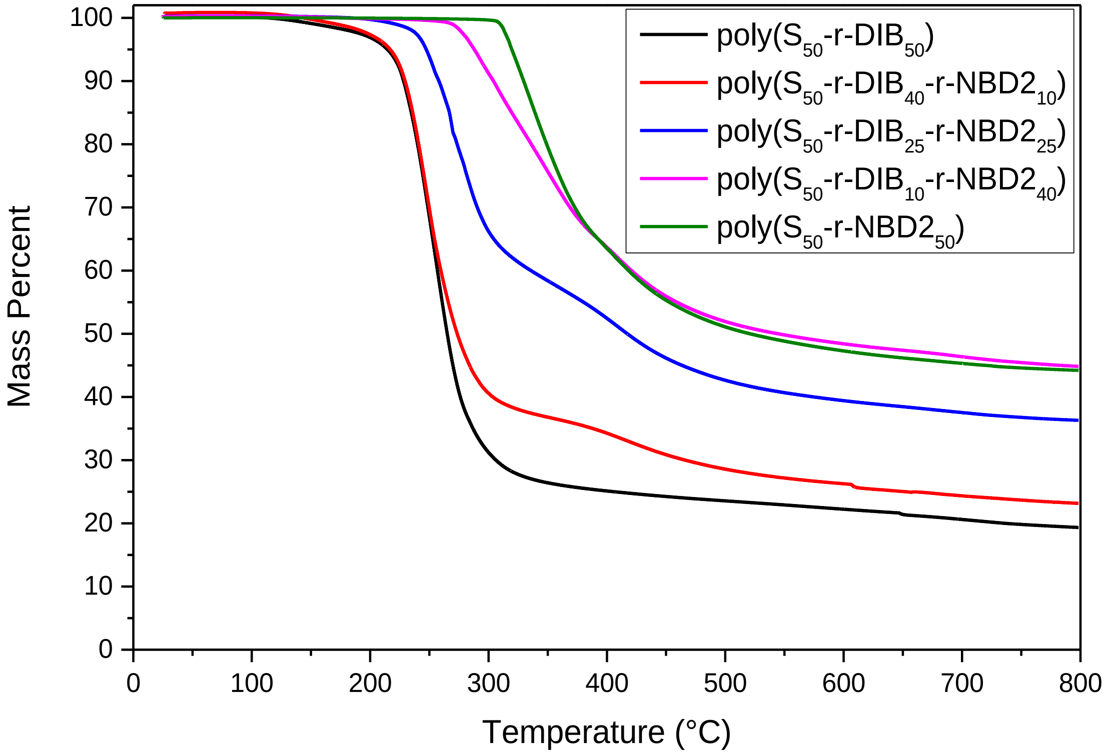

Supplementary Figure 5: TGA Results. Thermograms of poly(S-r-DIB) and poly(S-r-NBD2) copolymers as well as poly(S-r-DIB-r-NBD2) terpolymers all with 50 wt% sulfur

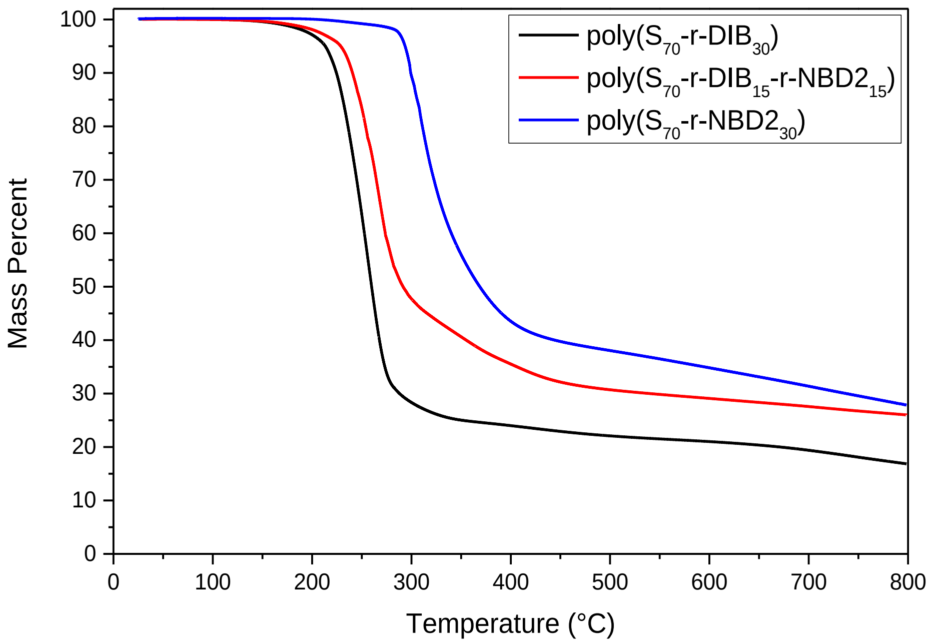

Supplementary Figure 6: TGA Results. Thermograms of poly(S-r-DIB) and poly(S-r-NBD2) copolymers as well as the poly(S-r-DIB-r-NBD2) terpolymer all with 70 wt% sulfur

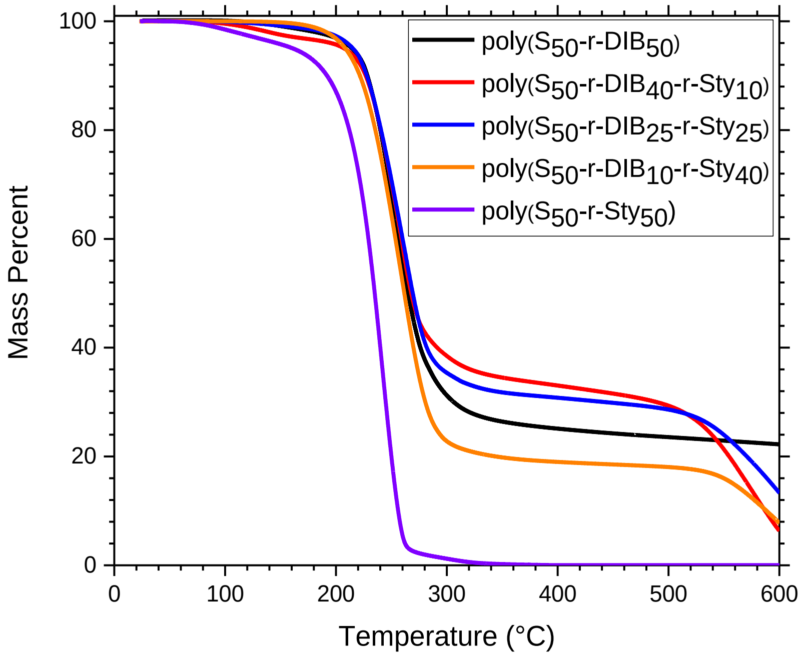

Supplementary Figure 7: TGA Results. Thermograms of poly(S-r-DIB) and poly(S-r-Sty) copolymers as well as poly(S-r-DIB-r-Sty) terpolymers all with 50 wt% sulfur

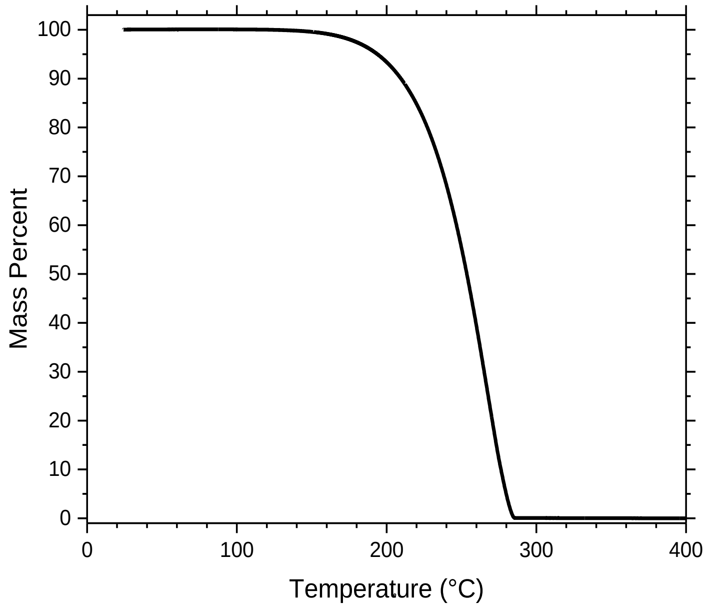

Supplementary Figure 8: TGA results. Thermogram of elemental sulfur showing a T_{deg}=194 °C

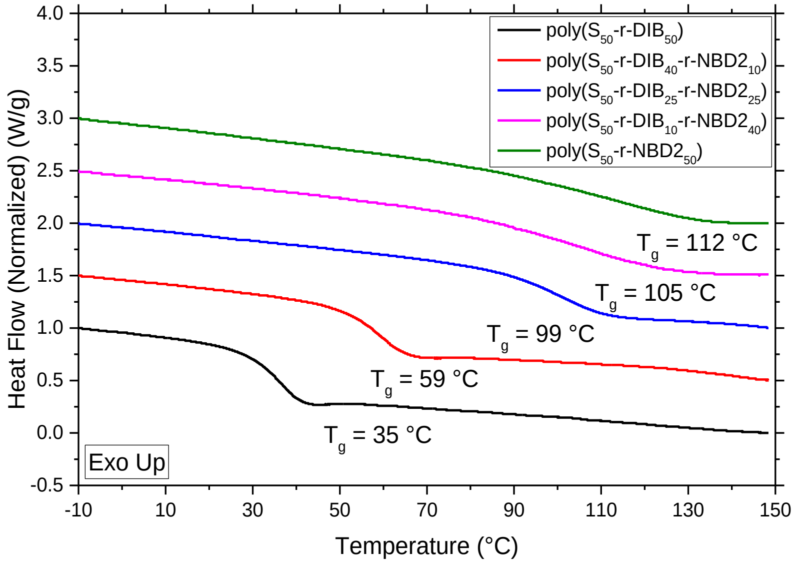

Supplementary Figure 9: DSC Results. Thermograms of poly(S-r-DIB) and poly(S-r-NBD2) copolymers as well as poly(S-r-DIB-r-NBD2) terpolymers all with 50 wt% sulfur

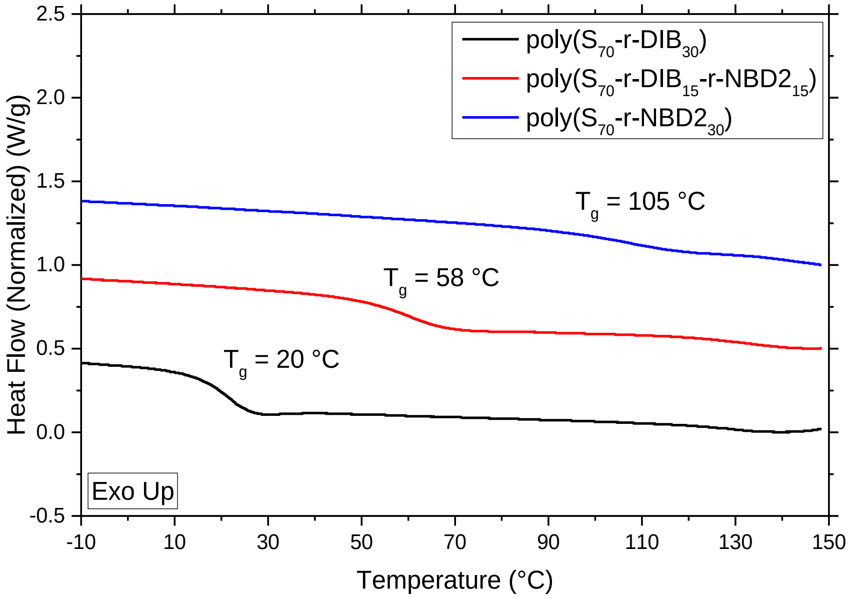

Supplementary Figure 10: DSC Results. Thermograms of poly(S-r-DIB) and poly(S-r-NBD2) copolymers as well as the poly(S-r-DIB-r-NBD2) terpolymer all with 70 wt% sulfur

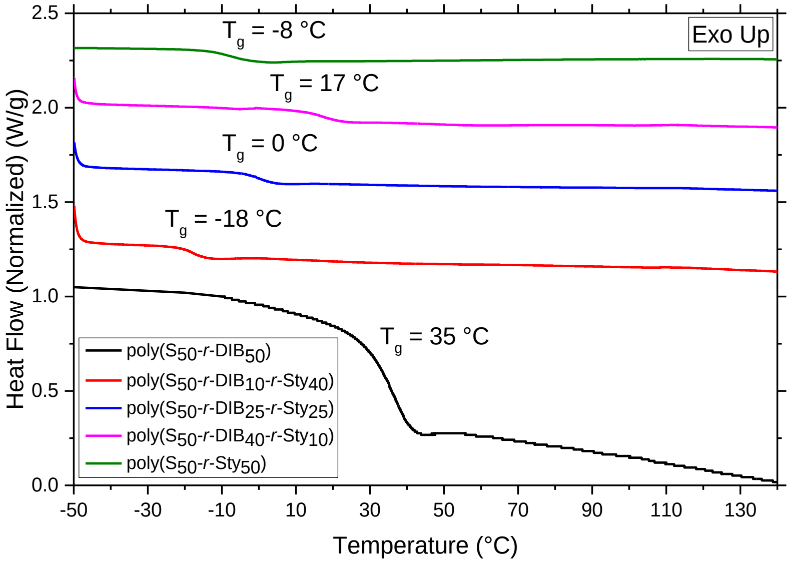

Supplementary Figure 11: DSC Results. Thermograms of poly(S-r-DIB) and poly(S-r-Sty) copolymers as well as poly(S-r-DIB-r-Sty) terpolymers all with 50 wt% sulfur

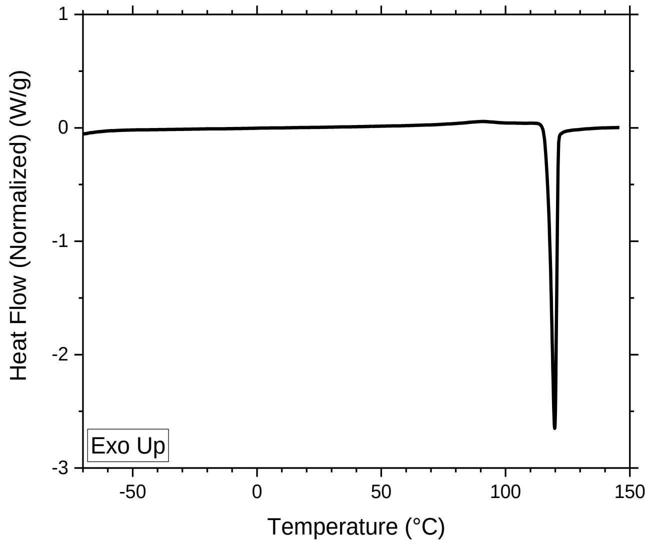

Supplementary Figure 12: DSC Results. Thermogram of elemental sulfur showing a T_m peak near 120 °C

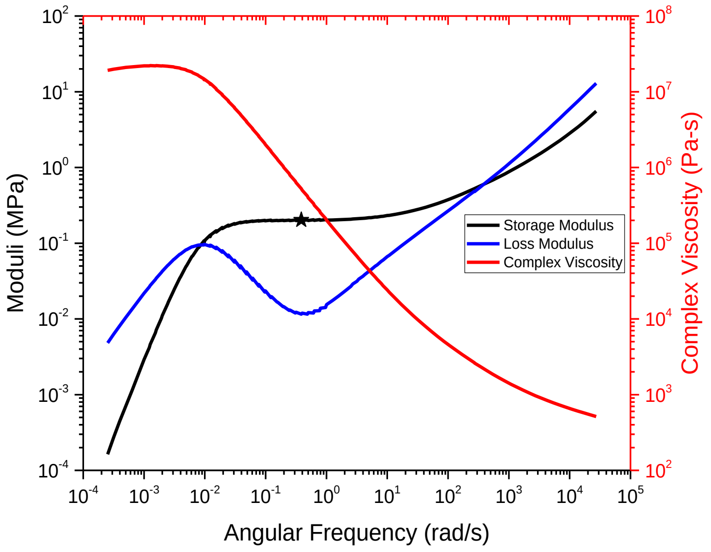

Supplementary Figure 13: Master curve of poly(S_{50}-r-DIB_{50}) at T_{ref}=100 °C. Master curve is constructed from standard TTS. The relaxation time at the crossover is $\tau = [8.56x10^{-3}\ rad/s]^{-1} = 116.8$ sec, the terminal viscosity is ~$2.19x10^7$ Pa-s, and the rubbery plateau modulus is $2.01x10^5$ Pa (black point marked by star).

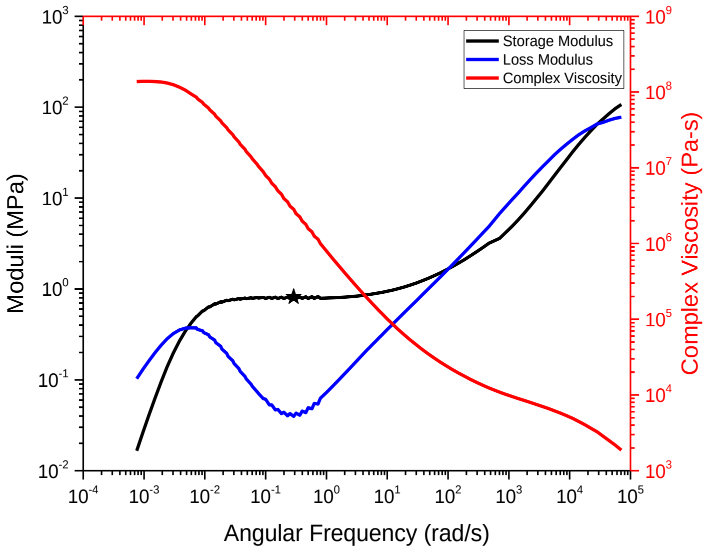

Supplementary Figure 14: Master curve of poly(S_{50}-r-DIB_{40}-r-$NBD2_{10}$) at T_{ref}=110 °C. Master curve is constructed from standard TTS. The relaxation time at the crossover is $\tau = [5.19x10^{-3}\ rad/s]^{-1} = 193$ sec, the terminal viscosity is ~$1.39x10^{8}$ Pa-s, and the rubbery plateau modulus is $8.13x10^{5}$ Pa (black point marked by star).

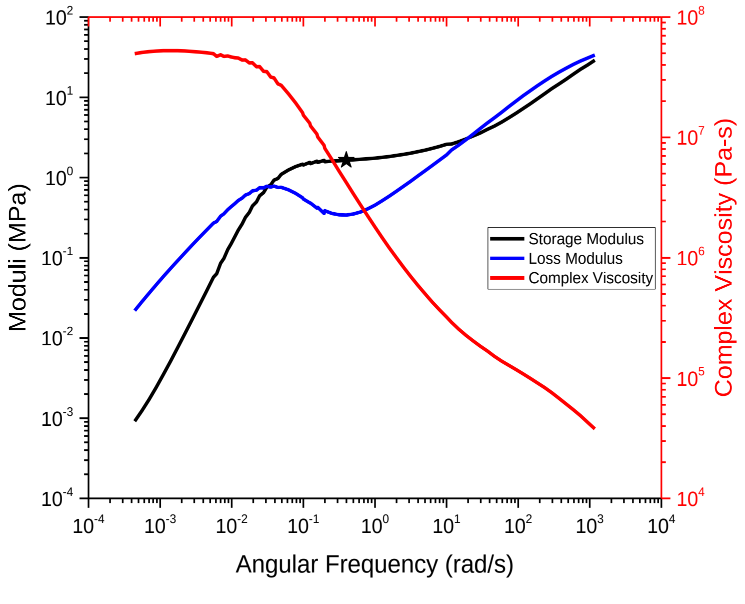

Supplementary Figure 15: Master curve of poly(S_{50}-r-DIB_{25}-r-$NBD2_{25}$) at T_{ref}=150 °C. Master curve is constructed from standard TTS. The relaxation time at the crossover is $\tau = [0.032\ rad/s]^{-1} = 31.3$ sec, the terminal viscosity is ~$5.22x10^{7}$ Pa-s, and the rubbery plateau modulus is $1.64x10^{6}$ Pa (black point marked by star).

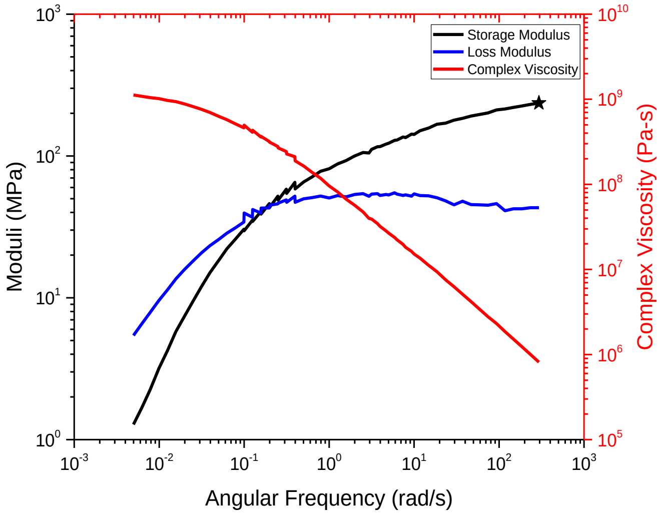

Supplementary Figure 16: Master curve of poly(S_{50}-r-DIB_{10}-r-$NBD2_{40}$) at T_{ref}=200 °C. Master curve is constructed from standard TTS. The relaxation time at the crossover is $\tau = [0.146\, rad/s]^{-1} = 6.85$ sec, the terminal viscosity is ~1.12×10^9 Pa-s, and the rubbery plateau modulus is 2.36×10^8 Pa (black point marked by star).

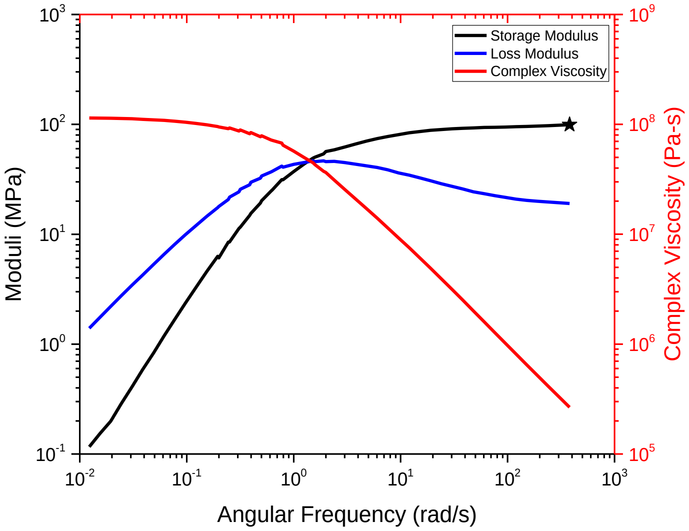

Supplementary Figure 17: Master curve of poly(S_{50}-r-$NBD2_{50}$) at T_{ref}=230 °C. Master curve is constructed from standard TTS. The relaxation time at the crossover is $\tau = [1.34\, rad/s]^{-1} = 0.747$ sec, the terminal viscosity is ~1.14×10^8 Pa-s, and the rubbery plateau modulus is 9.93×10^7 Pa (black point marked by star).

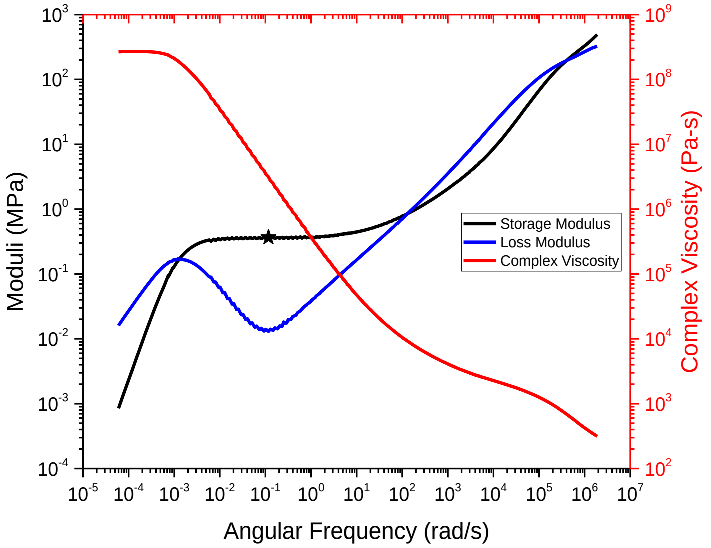

Supplementary Figure 18: Master curve of poly(S_{70}-r-DIB_{30}) at T_{ref}=90 °C. Master curve is constructed from standard TTS. The relaxation time at the crossover is $\tau = [1.30x10^{-3}\ rad/s]^{-1} = 769$ sec, the terminal viscosity is ~2.67x10^8 Pa-s, and the rubbery plateau modulus is 3.67x10^5 Pa (black point marked by star).

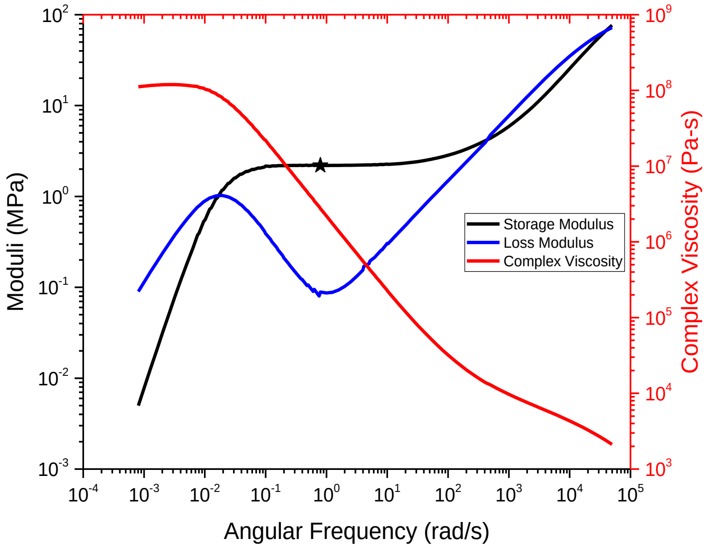

Supplementary Figure 19: Master curve of poly(S_{70}-r-DIB_{15}-r-$NBD2_{15}$) at T_{ref}=120 °C. Master curve is constructed from standard TTS. The relaxation time at the crossover is $\tau = [1.69x10^{-2}\ rad/s]^{-1} = 59.2$ sec, the terminal viscosity is ~1.19x10^8 Pa-s, and the rubbery plateau modulus is 2.19x10^6 Pa (black point marked by star).

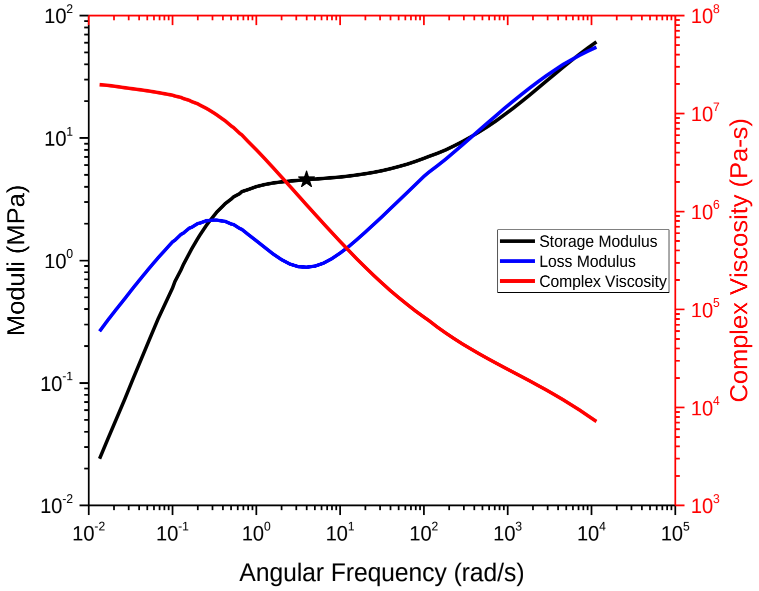

Supplementary Figure 20: Master curve of poly(S_{70}-r-$NBD2_{30}$) at T_{ref}=160 °C. Master curve is constructed from standard TTS. The relaxation time at the crossover is $\tau = [0.281\ rad/s]^{-1} = 3.56$ sec, the terminal viscosity is ~1.9x10^7 Pa-s, and the rubbery plateau modulus is 4.57x10^6 Pa (black point marked by star).

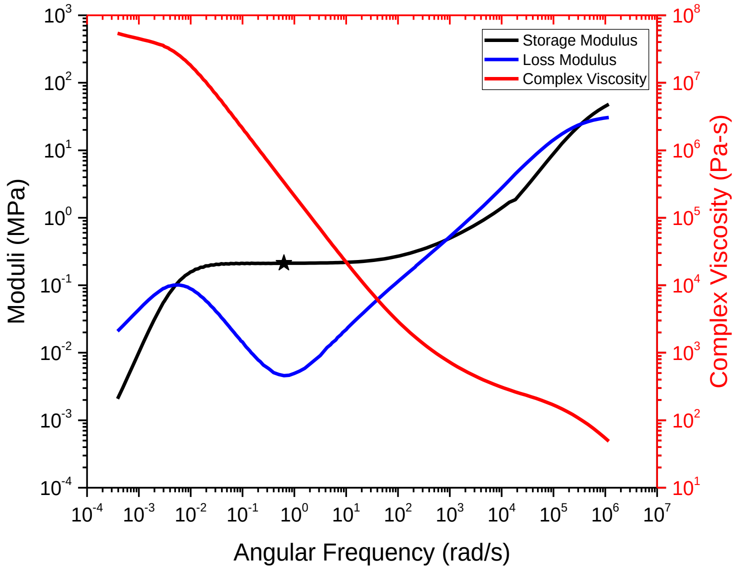

Supplementary Figure 21: Master curve of poly(S_{50}-r-DIB_{40}-r-Sty_{10}) at T_{ref}=90 °C. Master curve is constructed from standard TTS. The relaxation time at the crossover is $\tau = [5.14x10^{-3}\ rad/s]^{-1} = 195$ sec, the terminal viscosity is ~5.40x10^7 Pa-s, and the rubbery plateau modulus is 2.12x10^5 Pa (black point marked by star).

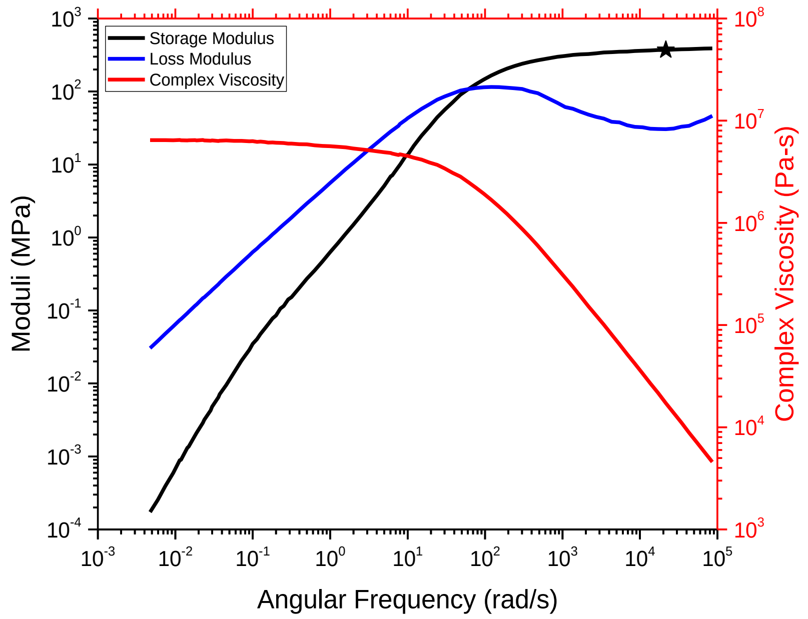

Supplementary Figure 22: Master curve of poly(S_{50}-r-DIB_{10}-r-Sty_{40}) at T_{ref}=10 °C. Master curve is constructed from standard TTS. The relaxation time at the crossover is $\tau = [61.1\ rad/s]^{-1} = 16.4$ msec, the terminal viscosity is ~6.45x10^6 Pa-s, and the rubbery plateau modulus is 371 MPa.

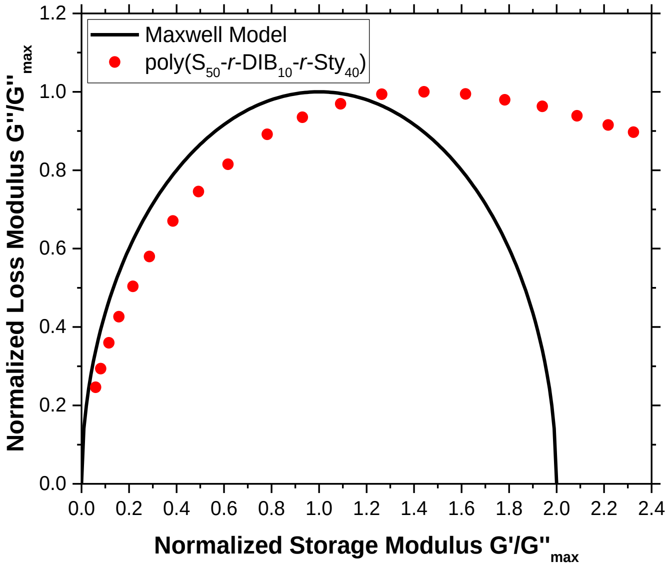

Supplementary Figure 23: Cole-Cole plot of poly(S_{50}-r-DIB_{10}-r-Sty_{40}). The material is not Maxwellian since the data is not semicircular.

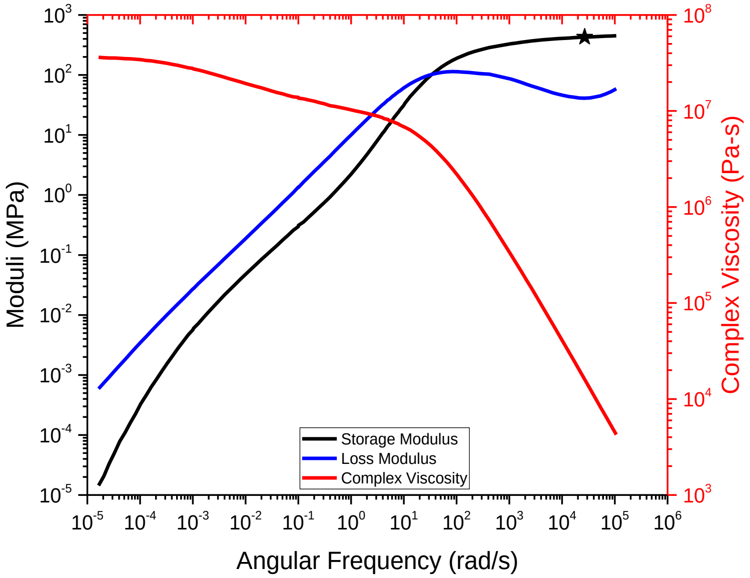

Supplementary Figure 24: Master curve of poly(S_{50}-r-DIB_{25}-r-Sty_{25}) at T_{ref}=20 °C. Master curve is constructed from standard TTS. The relaxation time at the crossover is $\tau = [34.1\ rad/s]^{-1} = 29.3$ msec, the terminal viscosity is ~3.62x10^7 Pa-s, and the rubbery plateau modulus is 427 MPa.

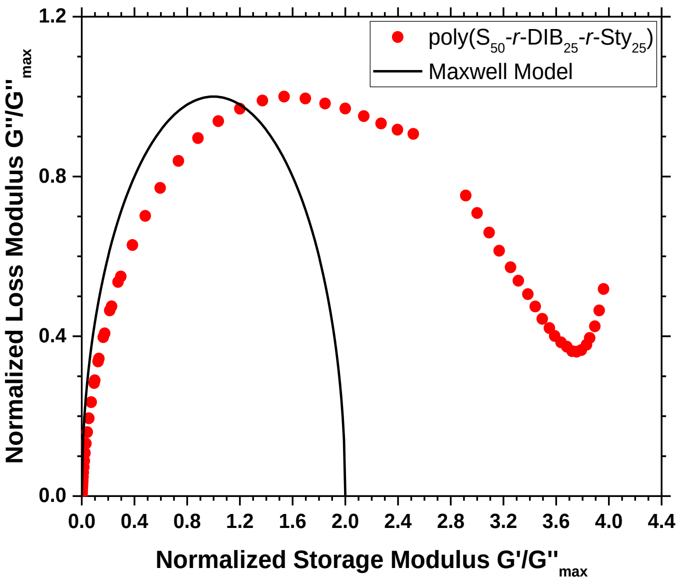

Supplementary Figure 25: Cole-Cole plot of poly(S_{50}-r-DIB_{25}-r-Sty_{25}) The material is not Maxwellian since the data is not semicircular.

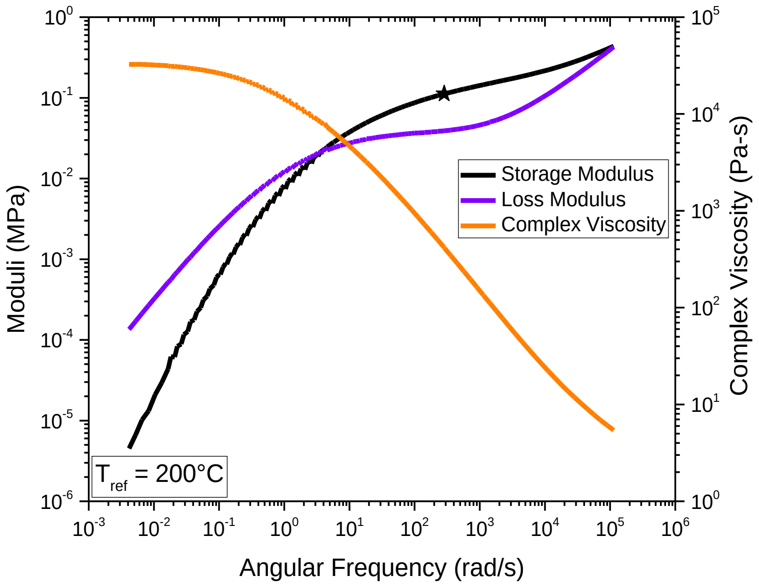

Supplementary Figure 26: Master curve of polystyrene at T_{ref}=200 °C. Master curve is constructed from standard TTS for comparison to a typical linear carbon-based polymer. The relaxation time at the crossover is $\tau = [3.55\ rad/s]^{-1} = 0.282$ sec, the terminal viscosity is ~3.25x10^4 Pa-s, and the rubbery plateau modulus is ~0.117 MPa.

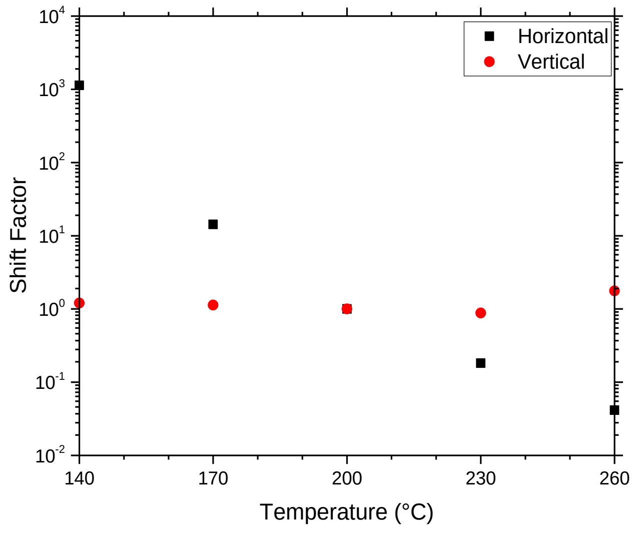

Supplementary Figure 27: Corresponding horizontal and vertical shift factors for the above polystyrene master curve. The horizontal shift factors vary over a similar order of magnitudes; however, the vertical shift factors are quite small, around one across the wider temperature change of 120 °C. In the calculation of vertical shift factor, the increase in temperature is largely balanced by the decrease in density and thus it remains near one.

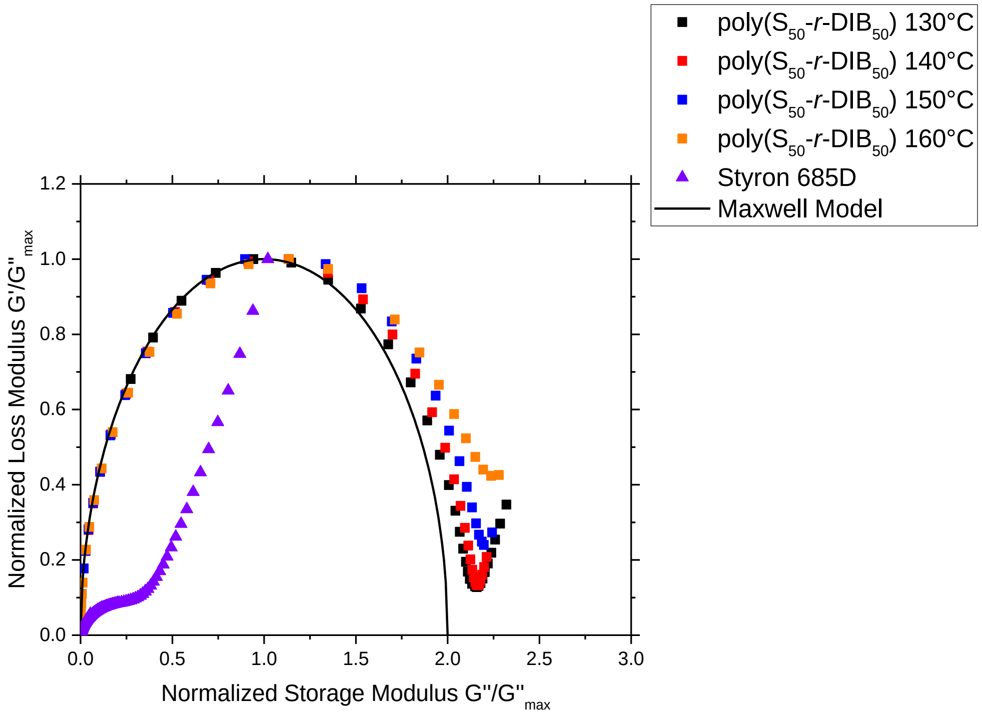

Supplementary Figure 28: Cole-Cole plot of polystyrene compared to poly(S_{50}-r-DIB_{50}). Polystyrene does not show Maxwellian behavior and is representative of most polymers.

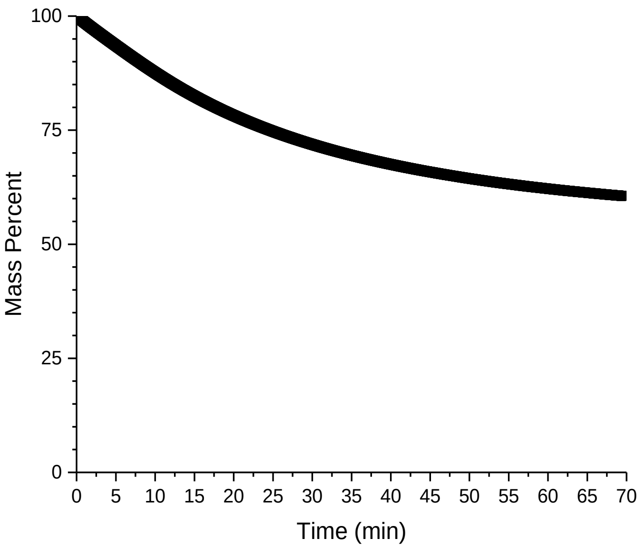

Supplementary Figure 29: TGA results. poly(S_{70}-r-DIB_{15}-r-$NBD2_{15}$) mass loss after ramping at 10 °C/min to 205 °C. Note that 4.62% mass was lost during the ramping.

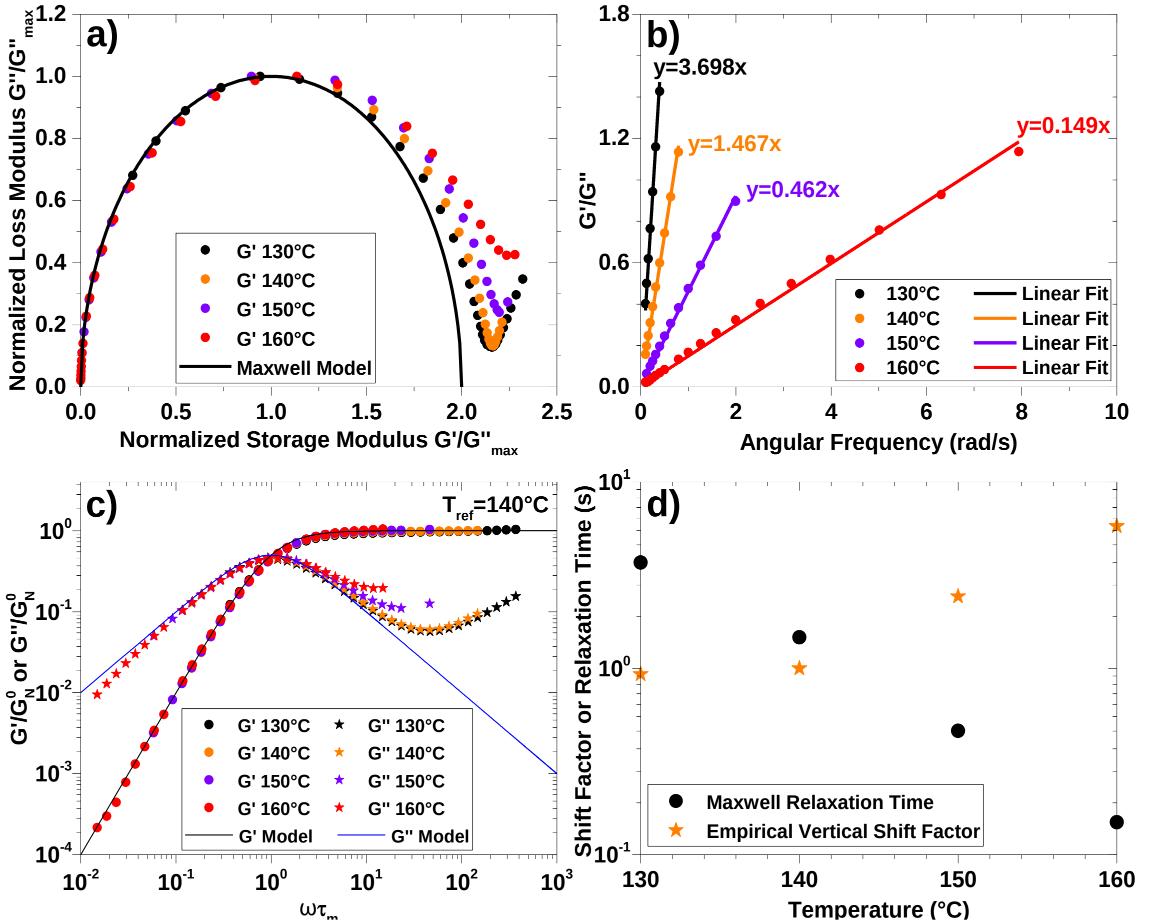

Supplementary Figure 30: Rheological results for poly(S_{50}-r-DIB_{50}) a) Cole-Cole plot showing the semicircular shape indicating that the material is Maxwellian and dominated by a single relaxation mode. b) Determination of the Maxwell relaxation time at each temperature displaying a maximum in loss modulus as predicted by the Maxwell model. c) Dimensionless master curve utilizing Maxwell relaxation times for horizontal shifting and empirical shifting along the vertical axis. Solid black and blue lines show the Maxwell model. d) Resulting shift factors showing the importance of the vertical shift factor

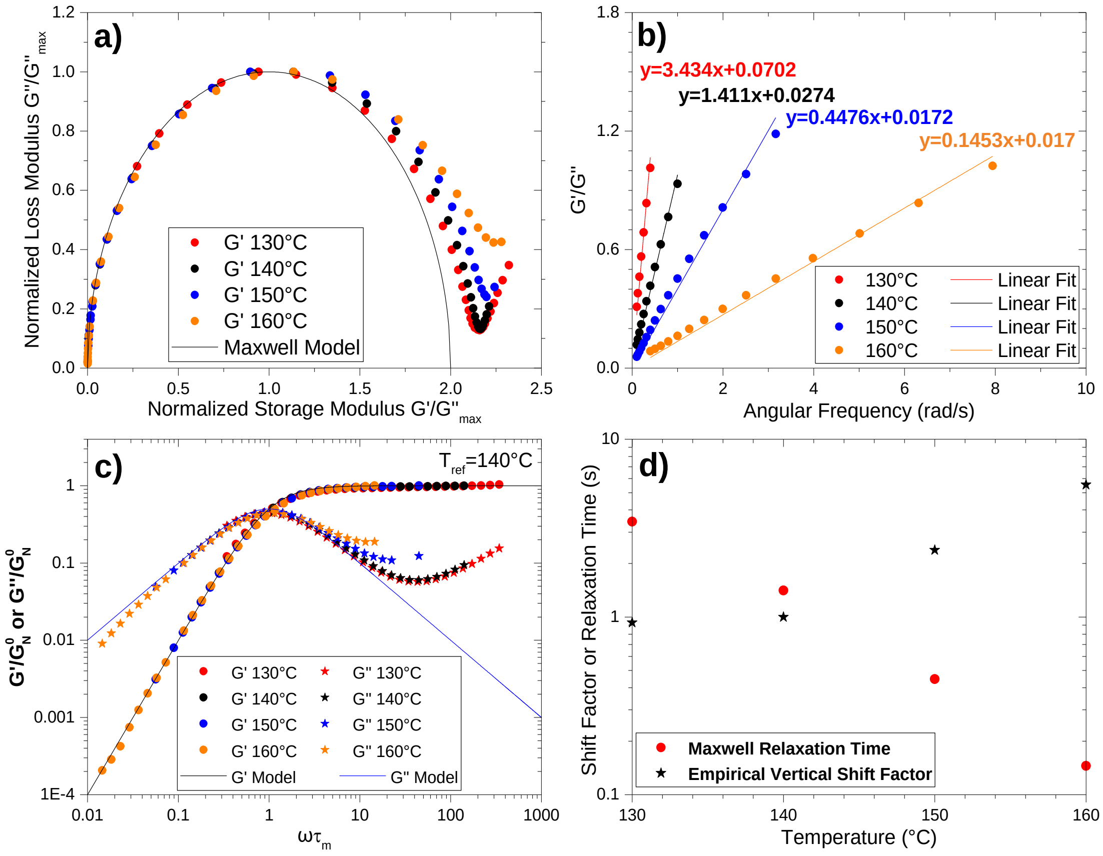

Supplementary Figure 31: Rheological results for poly(S_{50}-r-DIB_{50}) using a linear fit to calculate Maxwell relaxation times with a nonzero intercept. a) Cole-Cole plot showing the semicircular shape indicating that the material is Maxwellian and dominated by a single relaxation mode. b) Determination of the Maxwell relaxation time at each temperature displaying a maximum in loss modulus as predicted by the Maxwell model. c) Dimensionless master curve utilizing Maxwell relaxation times for horizontal shifting and empirical shifting along the vertical axis. Solid black and blue lines show the Maxwell model. d) Resulting shift factors showing the importance of the vertical shift factor

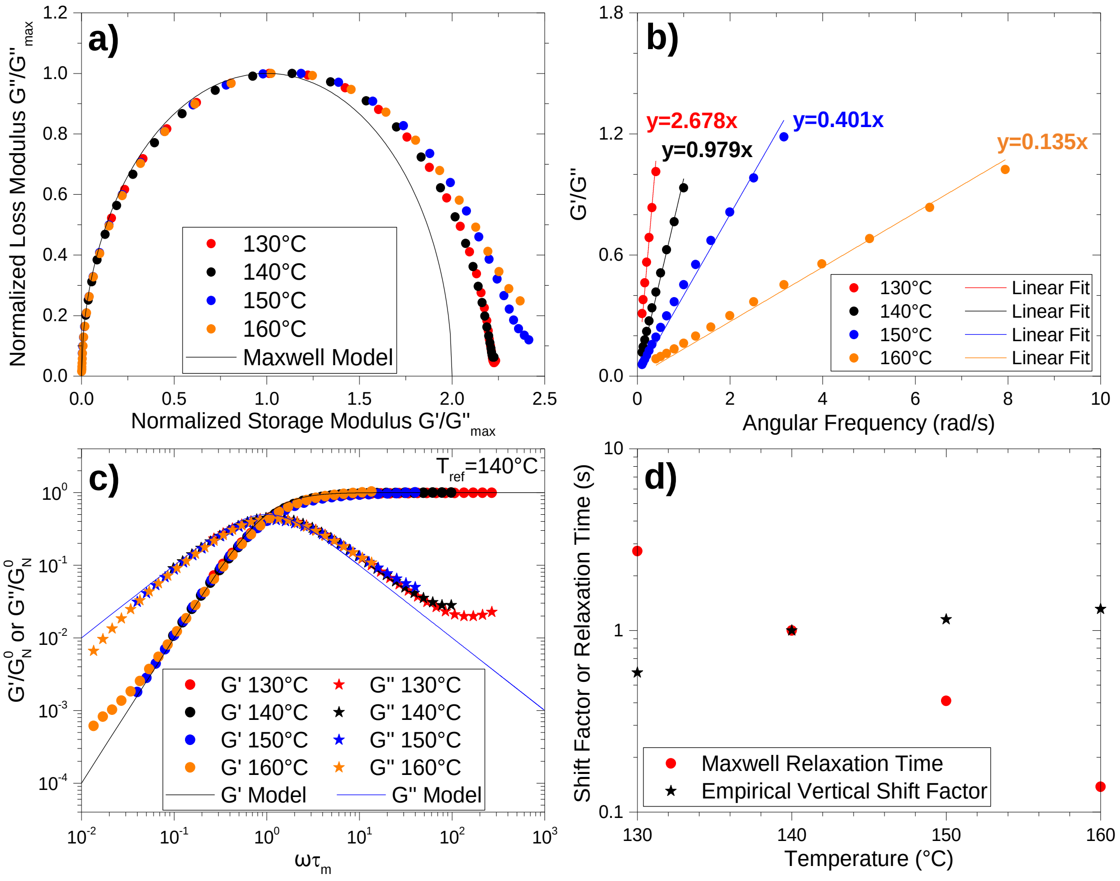

Supplementary Figure 32: Rheological results for poly(S_{50}-r-DIB_{50}) using new material for each frequency sweep. It was hypothesized that deviations in the loss modulus at higher frequencies (Fig. 4c in main text) were caused by sulfur subliming from the material (since sulfur is soluble in the polymer and is difficult to separate) or from sulfur bloom leading to sublimation over extended periods of elevated temperature exposure (Supplementary Fig. S17). a) Cole-Cole plot showing the semicircular shape indicating that the material is Maxwellian and dominated by a single relaxation mode. b) Determination of the Maxwell relaxation time at each temperature displaying a maximum in loss modulus as predicted by the Maxwell model. c) Dimensionless master curve utilizing Maxwell relaxation times for horizontal shifting and empirical shifting along the vertical axis. Solid black and blue lines show the Maxwell model. d) Resulting shift factors showing the importance of the vertical shift factor

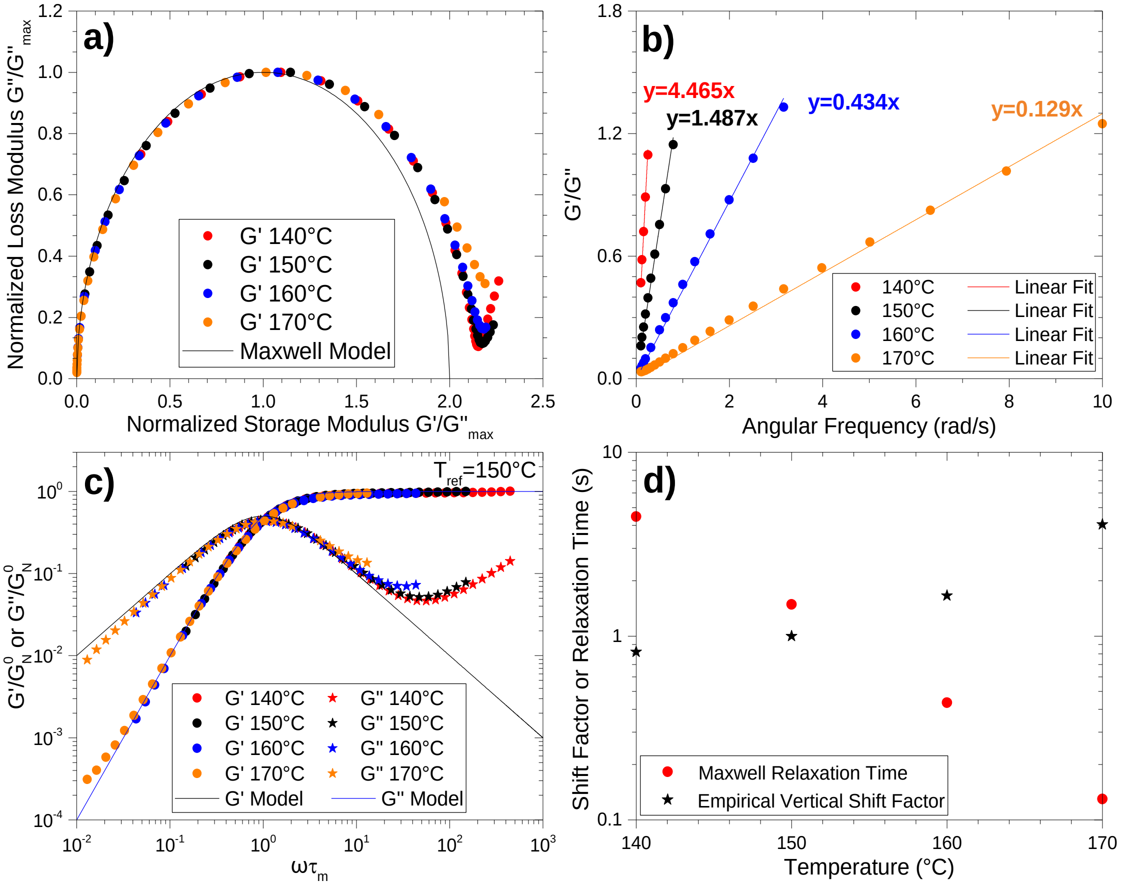

Supplementary Figure 33: Rheological results for poly(S_{50}-r-DIB_{40}-r-$NBD2_{10}$). a) Cole-Cole plot showing the semicircular shape indicating that the material is Maxwellian and dominated by a single relaxation mode. b) Determination of the Maxwell relaxation time at each temperature displaying a maximum in loss modulus as predicted by the Maxwell model. c) Dimensionless master curve utilizing Maxwell relaxation times for horizontal shifting and empirical shifting along the vertical axis. Solid black and blue lines show the Maxwell model. d) Resulting shift factors showing the importance of the vertical shift factor

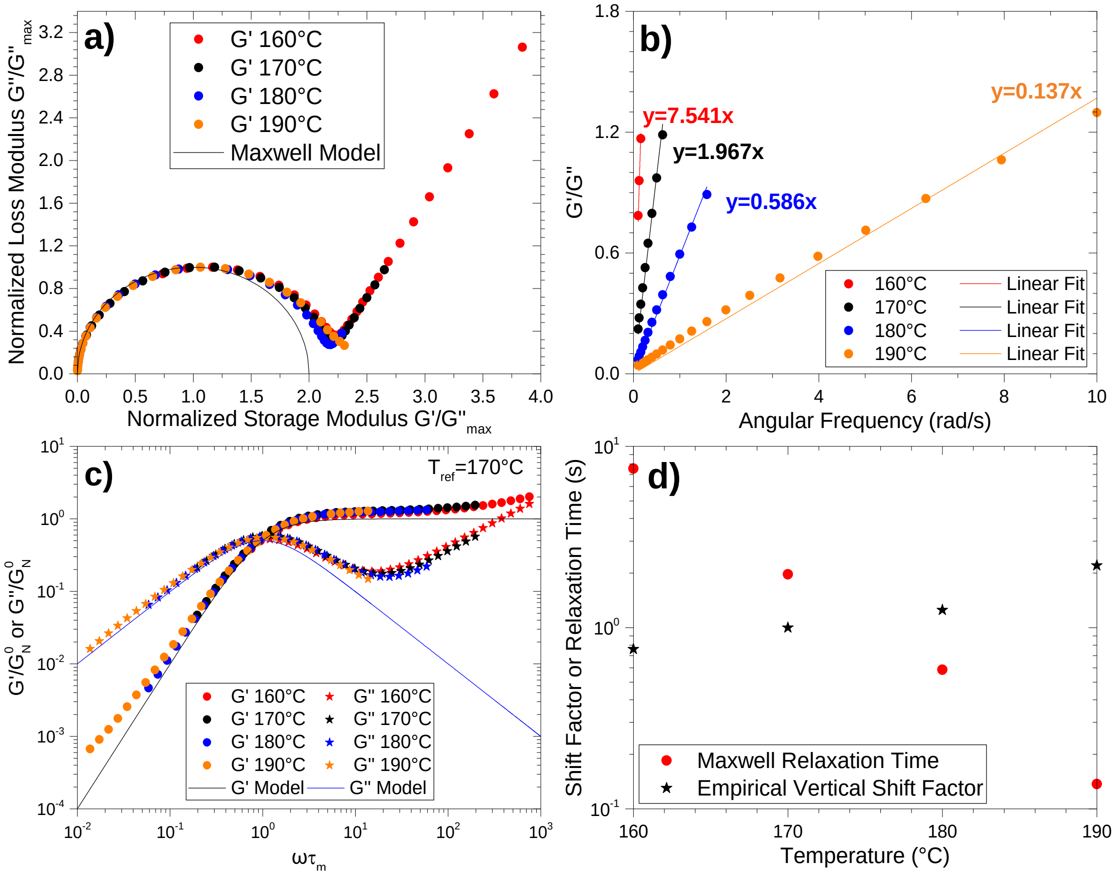

Supplementary Figure 34: Rheological results for poly(S_{50}-r-DIB_{25}-r-$NBD2_{25}$). a) Cole-Cole plot showing the semicircular shape indicating that the material is Maxwellian and dominated by a single relaxation mode. b) Determination of the Maxwell relaxation time at each temperature displaying a maximum in loss modulus as predicted by the Maxwell model. c) Dimensionless master curve utilizing Maxwell relaxation times for horizontal shifting and empirical shifting along the vertical axis. Solid black and blue lines show the Maxwell model. d) Resulting shift factors showing the importance of the vertical shift factor

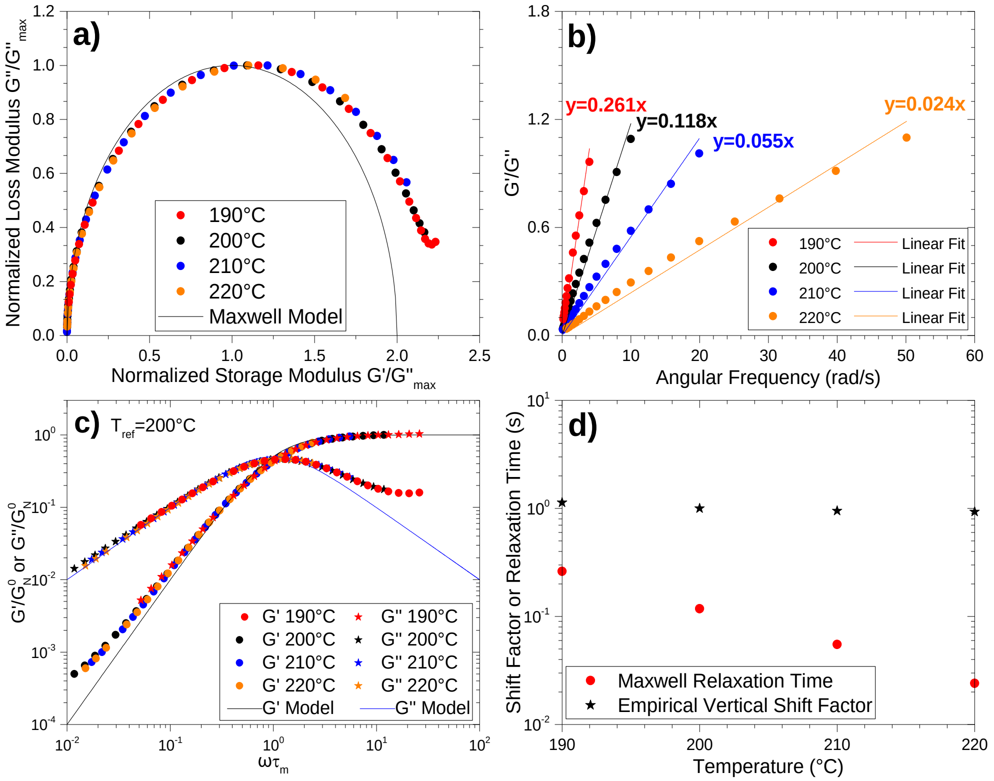

Supplementary Figure 35: Rheological results for poly(S_{70}-r-$NBD2_{30}$). a) Cole-Cole plot showing the semicircular shape indicating that the material is Maxwellian and dominated by a single relaxation mode. b) Determination of the Maxwell relaxation time at each temperature displaying a maximum in loss modulus as predicted by the Maxwell model. c) Dimensionless master curve utilizing Maxwell relaxation times for horizontal shifting and empirical shifting along the vertical axis. Solid black and blue lines show the Maxwell model. d) Resulting shift factors showing the importance of the vertical shift factor

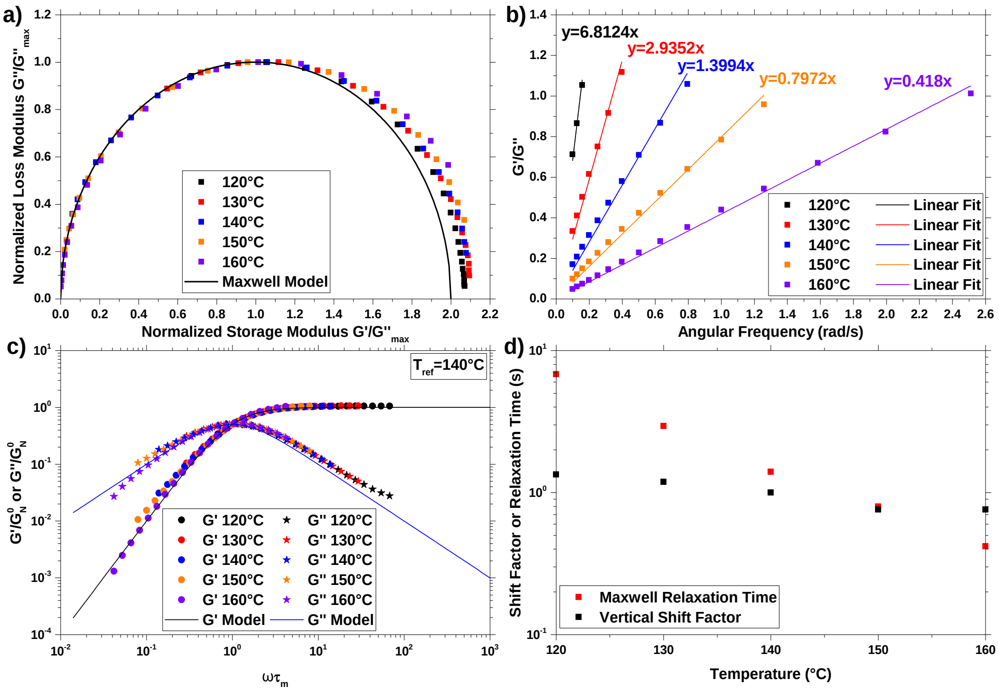

Supplementary Figure 36: Rheological results for poly(S_{50}-r-DIB_{40}-r-Sty_{10}). a) Cole-Cole plot showing the semicircular shape indicating that the material is Maxwellian and dominated by a single relaxation mode. b) Determination of the Maxwell relaxation time at each temperature displaying a maximum in loss modulus as predicted by the Maxwell model. c) Dimensionless master curve utilizing Maxwell relaxation times for horizontal shifting and empirical shifting along the vertical axis. Solid black and blue lines show the Maxwell model. d) Resulting shift factors showing the importance of the vertical shift factor

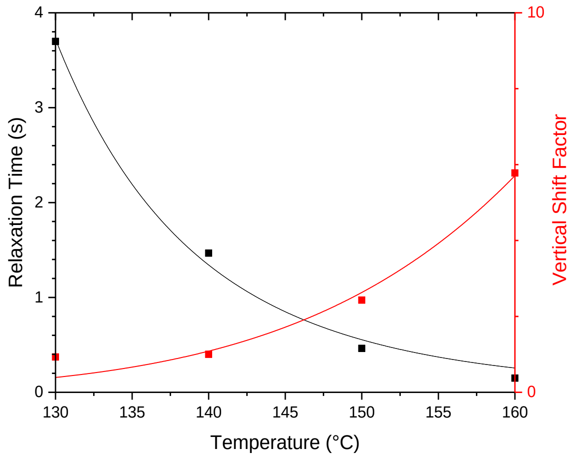

Supplementary Figure 37: Determination of activation energy. Arrhenius fits to both relaxation times and vertical shift factors for poly(S_{50}-r-DIB_{50}).

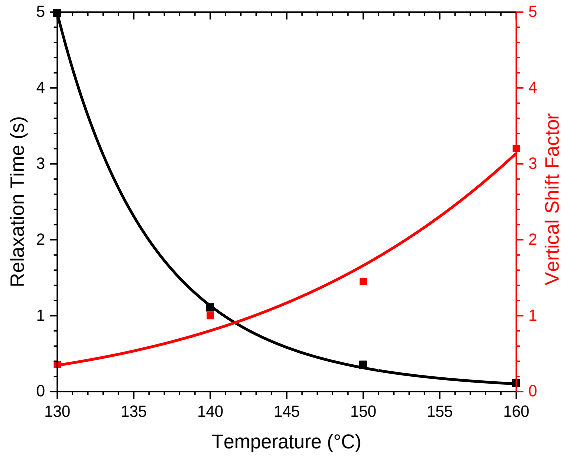

Supplementary Figure 38: Determination of activation energy. Arrhenius fits to both relaxation times and vertical shift factors for poly(S_{70}-r-DIB_{30}).

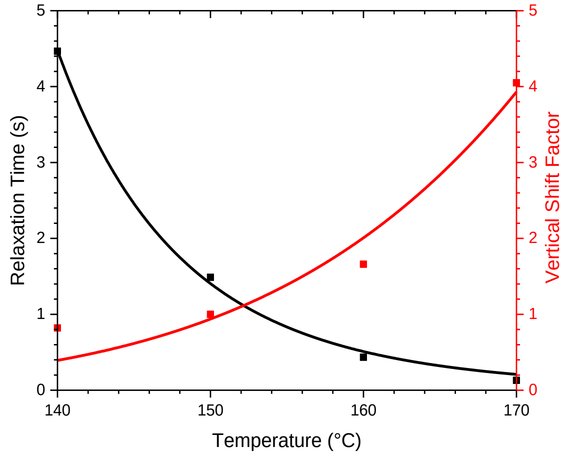

Supplementary Figure 39: Determination of activation energy. Arrhenius fits to both relaxation times and vertical shift factors for poly(S_{50}-r-DIB_{40}-r-$NBD2_{10}$).

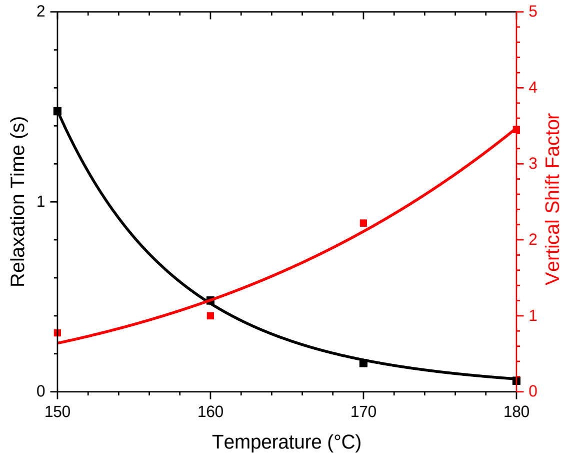

Supplementary Figure 40: Determination of activation energy. Arrhenius fits to both relaxation times and vertical shift factors for poly(S_{70}-r-DIB_{15}-r-$NBD2_{15}$).

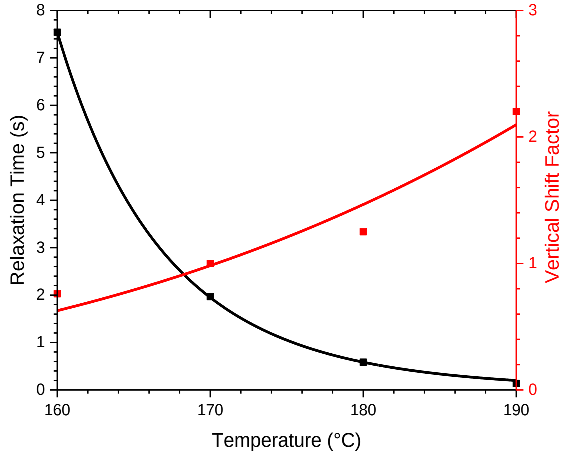

Supplementary Figure 41: Determination of activation energy. Arrhenius fits to both relaxation times and vertical shift factors for poly(S_{50}-r-DIB_{25}-r-$NBD2_{25}$).

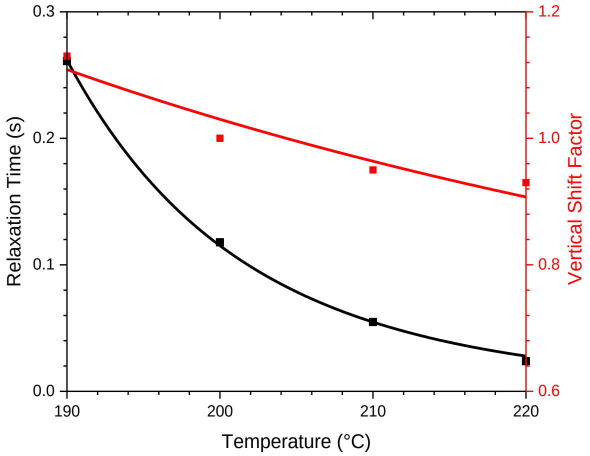

Supplementary Figure 42: Determination of activation energy. Arrhenius fits to both relaxation times and vertical shift factors for poly(S_{70}-r-NBD2$_{30}$).

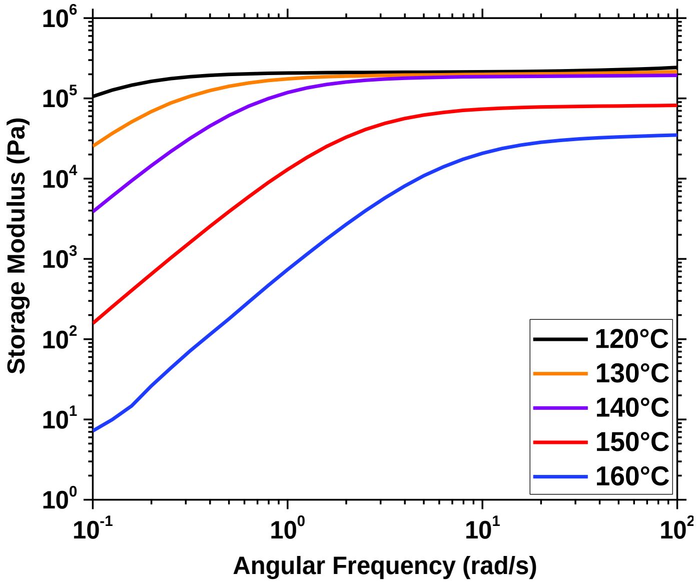

Supplementary Figure 43: Terminal region of Maxwellian copolymer poly(S_{50}-r-DIB_{50}). The drop off in storage modulus, here due to the oligopolysulfide exchange reaction, is a feature of covalent adaptable networks (CANs) in general that show a more rapid stress relaxation as the rate of reaction increases with temperature, thus shortening the rubbery plateau region. This unshifted data also emphasizes the importance of the vertical shift factor as its value increases as the rate of bond exchange increases.

Material	E_a from Maxwell Relaxation Times (kJ/mol)	Pre-exponential Factor (s)	E_a from Vertical Shift Factors (kJ/mol)	Pre-exponential Factor	Slope of $\log(b_T)$ vs. $\log(\tau_m)$
poly(S_{50}-r-DIB_{50})	15.4	2.3×10^{-6}	15.5	6.35×10^{5}	0.623
poly(S_{50}-r-DIB_{40}-r-$NBD2_{10}$)	20.2	1.2×10^{-7}	15.2	1.83×10^{5}	0.685
poly(S_{50}-r-DIB_{25}-r-$NBD2_{25}$)	30.6	7.7×10^{-10}	10.2	1.31×10^{3}	0.806
poly(S_{70}-r-DIB_{30})	22.4	4.8×10^{-9}	12.7	4.43×10^{4}	0.637
poly(S_{70}-r-DIB_{15}-r-$NBD2_{15}$)	23.1	1.3×10^{-8}	12.7	1.64×10^{4}	0.673
poly(S_{70}-r- $NBD2_{30}$)	25.9	1.9×10^{-8}	2.3	0.25	1.05

Supplementary Table 1: Evaluation of horizontal and vertical shift factor activation energies. Comparison of absolute values of activation energies from an Arrhenius fit of both Maxwell relaxation time and vertical shift factor versus temperature. The last column shows the absolute value of the slope between the temperature dependence of the shift factors giving insight into the underlying mechanism determining their values.
